# Effect of camel milk on lipid profile among patients with diabetes: a systematic review, meta-analysis, and meta-regression of randomized controlled trials

**DOI:** 10.1186/s12906-023-04257-5

**Published:** 2023-12-04

**Authors:** Narmin Khalid, Dana N. Abdelrahim, Nivine Hanach, Refat AlKurd, Moien Khan, Lana Mahrous, Hadia Radwan, Farah Naja, Mohamed Madkour, Khaled Obaideen, Husam Khraiwesh, MoezAlIslam Faris

**Affiliations:** 1 Department of Nutrition and Dietetics, Bahrain Defense Force Royal Medical Services Hospital, Riffa, Bahrain; 2 Sharjah Institute of Medical and Health Sciences (RIMHS), University of Sharjah, Sharjah, UAE; 3 Care and Public Health Research Institute (CAPHRI), Maastricht University, Maastricht, 6211 LM The Netherlands; 4 Department of Nutrition, Faculty of Pharmacy and Medical Sciences, University of Petra, Amman, Jordan; 5 Nutrition Studies Research Group, Department of Family Medicine, College of Medicine and Health Sciences, United Arab Emirates University, Al-Ain, UAE; 6 Primary Care, NHS Northwest London, TW3 3EB, London, UK; 7 Department of Health Sciences / Track of Clinical Nutrition, College of Health and Rehabilitation, Princess Nourah Bint Abdulrahman University, Riyadh, Saudi Arabia; 8 Department of Clinical Nutrition and Dietetics, College of Health Sciences, Sharjah Institute of Medical and Health Sciences (RIMHS), University of Sharjah, Sharjah, UAE; 9 Department of Medical Laboratory Sciences, College of Health Sciences, University of Sharjah, Sharjah, UAE; 10 Center for Advanced Materials Research, University of Sharjah, Sharjah, UAE; 11 Department of Nutrition and Food Processing, College of Agricultural Technology, Al-Balqa University, Salt, Jordan

**Keywords:** *Camelus dromedarius*, Arabian camel, Complementary and alternative medicine (CAM), Cardiometabolic markers, Dyslipidemia, Hypercholesterolemia, Milk

## Abstract

The effects of camel milk (CM) intake on lipid profile among patients with diabetes remain controversial. This systematic review and meta-analysis of randomized controlled trials (RCTs) aimed to calculate the effect size of CM intake on blood lipids among patients with type 1 (T1D) and type 2 (T2D) diabetes. We searched nine databases from inception until December 31, 2022, to identify relevant RCTs. Effect sizes for total cholesterol (TC), triglycerides (TG), low-density lipoprotein (LDL), very low-density lipoprotein (VLDL), and high-density lipoprotein (HDL) were calculated and expressed using mean differences (MD) and confidence intervals (CI). Of 4,054 retrieved articles, 10 RCTs (a total of 347 participants aged 8–70 years, 60.5% male) were eligible for inclusion. The pooled results from a random-effects model showed statistically significant decreases in TC (MD − 21.69, 95% CI: 41.05, − 2.33; *p* = 0.03; *I*^2^=99%), TG (MD − 19.79, 95% CI: −36.16, − 3.42; *p*=0.02, *I*^2^=99%), and LDL (MD −11.92, CI: −20.57, −3.26; *p* = 0.007, *I*^2^=88%), and a significant increase in HDL (MD 10.37, 95% CI, 1.90, 18.84; *p*=0.02, *I*^2^=95%) in patients with diabetes supplemented with CM compared with usual care alone. Subgroup analysis revealed that only long-term interventions (> 6 months) elicited a significant reduction in TC levels and TG levels. Consumption of fresh CM by patients with diabetes resulted in significant reductions in TC, TG, and LDL levels, while showing a significant increase in HDL levels. Patients with T1D elicited a more beneficial effect in lowering TC, LDL, and TG levels and in increasing HDL levels than their corresponding partners with T2D. In conclusion, long-term consumption of CM for patients with diabetes, especially those with T1D, could be a useful adjuvant therapy to improve lipid profile alongside prescribed medications. However, the high heterogeneity in the included studies suggests that more RCTs with larger sample sizes and longer intervention durations are required to improve the robustness of the available evidence.

**Supplementary Information:**

The online version contains supplementary material available at 10.1186/s12906-023-04257-5.

## Introduction

Diabetes remains a substantial public health issue, with more than 1·31 billion (1·22–1·39) people projected to have diabetes by 2050 [1]. Diabetes is a major cardiometabolic risk factor that increases the likelihood of developing cardiovascular disease (CVD) [2]. People with diabetes are at higher risk for heart disease, stroke, high blood pressure, and other cardiovascular problems [3]. However, effective diabetes management can help reduce the risk of developing CVD [4].

Given the chronic nature of diabetes and the difficulties associated with adhering to its management protocol, numerous forms and modalities of complementary and alternative medicine (CAM) have been proposed to help control the negative sequelae of diabetes, including micro and macrovascular consequences [5, 6]. Extensive research has been conducted on the management of diabetes using hypoglycemic medications, insulin, and dietary interventions. Throughout history, a diverse range of traditional food therapies have been employed in the management of diabetes and the mitigation of associated problems [5]. In recent times, there has been a significant amount of research conducted on CAM in the context of managing diabetes [6]. Individuals diagnosed with type 2 diabetes (T2D) are motivated to effectively navigate the intricacies of their ailment, optimize their well-being, and mitigate associated problems by employing CAM modalities [6].

Camel milk, from *Camelus dromedarius*, is one of the most commonly utilized CAM therapies for diabetes in the Middle East, including the UAE [7, 8], especially with the escalating prevalence of diabetes in this region [9, 10]. Research has demonstrated that CM has a significant beneficial impact on human nutrition and health, and can be part of CAM because of its multiple functional qualities relevant to the prevention and treatment of many acute and chronic diseases [11–16]. In particular, raw CM has been used as an alternative to current treatments for lipid abnormalities such as dyslipidemia linked to diabetes and other health conditions [17–21]. Currently, CM is the fifth source of milk in the global market, with about 3,200 million liters produced each year [22]. The results of the chemical analysis indicate that CM exhibits a lower content of cholesterol and saturated fats and a greater content of unsaturated fats in comparison to cow’s milk [23]. As a result, CM may possess a potential advantage over cow’s milk in terms of its ability to normalize lipid profile, which is a significant determinant in enhancing cardiometabolic health [16, 24–28]. Camel milk and its protein hydrolysates have been found to confer bio-functionalities, including antioxidant, antimicrobial, antidiabetic, antiradical, angiotensin-converting enzyme inhibiting, anti-inflammatory, anti-cancer, anti-allergic, hepatoprotective, and anti-autism properties [29, 30]. The most influential bioactive chemicals in CM include minerals (e.g., Mg and Zn), vitamins (e.g., E and C), protective proteins (e.g., lysozyme, lactoferrin, and immunoglobulin), and antioxidant enzymes (e.g., superoxide dismutase and glutathione peroxidase) [29, 30].

Recent clinical trials by Sboui et al. (2022) [31] and Zheng et al. (2021) [32] revealed consumption of CM by patients with T2D significantly improved their lipid profile, particularly in lowering serum total cholesterol (TC) and triglycerides (TG). However, an earlier clinical trial that examined the impact of CM on the lipid profiles of patients with diabetes found no significant changes in lipid profile [33] compared with the control group. These results highlighted the controversy regarding the impact of CM consumption on lipid profiles among patients with diabetes. The small number of available studies combined with a lack of quantitative assessment means it is difficult to ascertain and determine the accurate effect of CM consumption on the lipid profile among patients with diabetes.

Despite some evidence that CM may have beneficial effects on lipid profiles among patients with diabetes, the potential benefits and risks of CM consumption in this population remain unclear. Therefore, the present meta-analysis aimed to offer a reliable estimate of the effect sizes of CM intake on lipid profiles among patients with diabetes, analyze the generalizability of findings implying CM as an effective remedy for diabetes, assess variations between studies, and perform subgroup analyses for key variables, such as type of disease (T1D or T2D), type of CM (fresh or fermented/pasteurized), and duration of CM intake (≤ 6 or > 6 months). Based on existing knowledge about CM, we hypothesized that compared with patients receiving standard customary care or other ruminant milk, intake of CM by patients with diabetes may improve their lipid profile, thereby improving their cardiometabolic health and reducing the risk for CVD.

## Materials and methods

We used the Preferred Reporting Items for Systematic Reviews and Meta-Analyses (PRISMA) as a guideline for reporting our findings [34]. The protocol for this study was registered with the International Prospective Register of Systematic Reviews (PROSPERO, CRD42021276157).

### Inclusion criteria

The inclusion criteria for intervention studies that examined the effect of CM intake on lipid profile were randomized controlled trials (RCTs) that: (1) involved patients with T1D or T2D; (2) included patients aged ≥18 years; (3) provided numerical data on the baseline and post-intervention measures of TC, TG, low-density lipoprotein (LDL), very-low-density lipoprotein (VLDL), and high-density lipoprotein (HDL) and among patients with diabetes receiving CM and control groups (i.e., patients with diabetes receiving conventional or standard therapy alone); and (4) were original research studies published in the English language.

### Exclusion criteria

To eliminate potential quality or methodological issues, we excluded: (1) non-experimental studies (case, longitudinal, cross-sectional, case-control, and cohort studies), editorials, observational abstracts, book chapters, letters to the editor, and literature reviews; (2) non-peer-reviewed and unpublished papers and non-English studies; (3) RCTs performed exclusively among healthy participants, children, athletes, lactating and pregnant women, and animals; (4) studies with insufficient numerical data reporting the study outcomes; and (5) studies reporting the presence of comorbidities with diabetes.

### Database search

Three authors (NK, DA, MF) conducted an electronic database search to locate relevant RCTs that assessed the impact of CM intake on lipid profiles among patients with diabetes. The search covered nine databases: CINAHL, Cochrane, Google Scholar, EBSCOhost, PubMed/MEDLINE, ScienceDirect, Web of Science, Scopus, and ProQuest. Databases were searched from inception (1950) until December 31, 2022. The search strategy included relevant key terms: “Camel milk” OR “dromedary camel milk” OR “Arabian *camel milk*” AND “diabetes” OR “diabetes” OR “type 1 diabetes” OR “T1D” OR “type 2 diabetes” OR “T2D” OR “juvenile diabetes” OR “adulthood diabetes” AND “lipid” OR “lipid profile” OR “Total cholesterol” OR “TC” OR “Triglycerides” OR “TG” OR “low-density lipoprotein” OR “LDL” OR “very low-density lipoprotein” OR “VLDL”, OR “high-density lipoprotein” OR “HDL”. The reference lists of retrieved studies and reviews were manually searched for additional relevant studies. Table 1 shows the comprehensive search approach.

**Table 1:** Summary of the search strategy used in this systematic review and meta-analysis that assessed the effects of camel milk intake on lipid profile among patients with diabetes

Search Strategy Item	Search Strategy Details
String of keywords	“Camel milk” OR “*dromedary* camel milk” OR “Arabian *camel milk*” AND “diabetes” OR “diabetes” OR “type 1 diabetes” OR “T1D” OR “type 2 diabetes” OR “T2D” OR “juvenile diabetes” OR “adulthood diabetes” AND “lipid” OR “lipid profile” OR “Total cholesterol” OR “Triglycerides” OR “HDL” OR “LDL” OR “VDL”
Searched databases	Google Scholar, PubMed/MEDLINE, EBSCOhost, CINAHL, ScienceDirect, Cochrane, ProQuest Medical, Web of Science, and Scopus
Inclusion criteria	P (People): All patients with diabetes (T1D, T2D), including males/females aged > 18 years, from unspecified ethnic/racial backgrounds I (Intervention/exposure): Intake of CM, in any form (fresh, dried/reconstituted, pasteurized, fermented/cultured) for any time duration C (Comparison): Comparing consumers with non-consumers of CM, routine, or usual diabetes care O (Outcome): Effect size of consuming CM on lipid profile in patients with diabetes, total cholesterol (TC), triglycerides (TG), high-density lipoprotein (HDL), low-density lipoprotein (LDL), very-low-density lipoprotein (VLDL) S (Study type): Original research, experimental/randomized controlled trial (RCT) study is eligible for inclusion
Exclusion criteria	P (People): Healthy, non-diabetic people, studies exclusively on children with diabetes, athletes, pregnant, lactating, animals, and patients with other comorbidities I (Intervention/Exposure): Non-CM C (Comparaison): Non-diabetes comparator O (Outcome): Outcomes not described in sufficient numerical detail for the lipid profile measures (using curves, and graphs without numerical presentations) S (Study type): Editorials, paper abstracts, book chapters, case reports, commentaries, expert opinions, letters to the editor, reviews, conference abstracts or proceedings; non-peer-reviewed and unpublished data
Moderators for meta-regression	Continuous, including the age of patients, time duration of CM intake (days/weeks/months); dichotomous, including sex (male/female) and type of diabetes (T1D, T2D), duration of CM intake (> 6 months, ≤ 6 months), type of CM (fermented/pasteurized, fresh)
Time filter	None applied (search from inception)
Language filter	English language only

### Main outcomes and measures

The primary outcome was the impact of CM intake on the lipid profile of patients with diabetes (i.e., TC, TG, LDL, VLDL, and HDL). To standardize data extraction, the review team collected and coded data for study characteristics (e.g., publication year, authors’ names, country/city, sample size, type of diabetes, type of CM, duration of supplementation with CM, the quantity of CM consumed per day) and participant characteristics (e.g., sex or proportion of male participants, age), as well as the key lipid profile findings in the control and intervention groups.

### Data extraction

Two authors (NK, and DA) screened the retrieved articles and extracted the data, and the other authors double-checked the extracted data. Disagreements were resolved by the chief investigator (MF). We developed a screening tool to extract data from each study that covered: participants’ sex and age, region of origin, first author’s name, publication year, sample size in each group, parameters measured, type of diabetes, type of CM, intervention duration, amount of CM consumed per unit time, mean and standard deviation (SD) for the outcome measures (control and intervention groups), and a summary of the significance of the results. Extracted data were entered into a Microsoft Excel spreadsheet in preparation for analysis.

### Quality assessment

The Cochrane Risk of Bias assessment tool was used to evaluate the included studies. This tool aims to improve the accuracy and clarity of bias assessment by examining six types of bias: selection bias, performance bias, reporting bias, detection bias, attrition bias, and other biases [35, 36]. Three authors scored the selected articles (MK, DA, LM), with any disagreements resolved by the principal investigator (MF).

### Data synthesis and statistical analysis

We used a meta-analysis random-effects model for all statistical tests, which assumed a distribution of true effect sizes rather than a single true effect size [37]. We estimated the mean of the genuine impact size distribution. Tau-square (*τ*^*2*^) was used to evaluate heterogeneity within studies, and *I*^*2*^ was used to evaluate heterogeneity between the included studies [38]. To ensure our meta-analysis results were not influenced by a single study, we performed a leave-one-out sensitivity analysis by deleting one study at a time. Computing *I*^*2*^ and *τ*^*2*^ statistics was important to examine heterogeneity [37, 38]. *I*^*2*^ values > 90% represent considerable heterogeneity, 60–90% represent substantial heterogeneity, 30–59% represent moderate heterogeneity, and < 30% represent low heterogeneity [38]. Graphical plots were used to visually aid the interpretation of the results [39]. Funnel plots were adopted to detect publication bias, and the nonparametric trim and fill technique was used to confirm the findings [40]. Finally, subgroup analyses were performed to evaluate differences in the effect of CM consumption between the primary factors reported as categorical variables (T1D or T2D, fresh or fermented/pasteurized CM, CM intake for ≤ 6 or > 6 months). Subgroup analyses were performed for cardiometabolic indicators that were reported in at least seven studies.

All effect sizes were represented as mean difference (MD) and 95% confidence interval (CI). The effect sizes were pooled using a random-effects model in RevMan software version 5.3.5 (The Nordic Cochrane Center, The Cochrane Collaboration, 2014). The mean net changes (mean, SD) for all variables between the CM intervention and control groups were calculated at the beginning and end of the trial. We calculated the SD using the formula from the Cochrane Handbook as follows.

From standard error when the SD was not given: $SD=SE\times \sqrt{N}$

When SD change was not given:

$${SD}_{E,change}=\sqrt{{SD}_{E,baseline}^{2}+{SD}_{E,final}^{2}-(2\times Corr\times {SD}_{E,baseline}\times {SD}_{E,final})}$$

When the combination of intervention groups was required:

$$\sqrt{\frac{\left({N}_{1}-1\right){SD}_{1}^{2}+\left({N}_{2}-1\right){SD}_{2}^{2}+\frac{{N}_{1}{N}_{2}}{{N}_{1}+{N}_{2}}({M}_{1}^{2}+{M}_{2}^{2}-2{M}_{1}{M}_{2})}{{N}_{1}+{N}_{2}-1}}$$

*I*^*2*^ was used to estimate heterogeneity between studies. The *I*^*2*^ statistic reflects the proportion of variance in effect estimates across studies that is attributable to heterogeneity as opposed to sampling error (I^2^ > 50%: considerable heterogeneity [41]). Probable publication bias was identified using funnel plots for the five tested parameters (Supplementary Figs. 1–5). A *p*-value < 0.05 was considered statistically significant. A sensitivity analysis excluding one study at a time was conducted to test the robustness of the overall findings and determine the effect of the results on the meta-analysis.

## Results

### Study selection

**Fig. 1:**
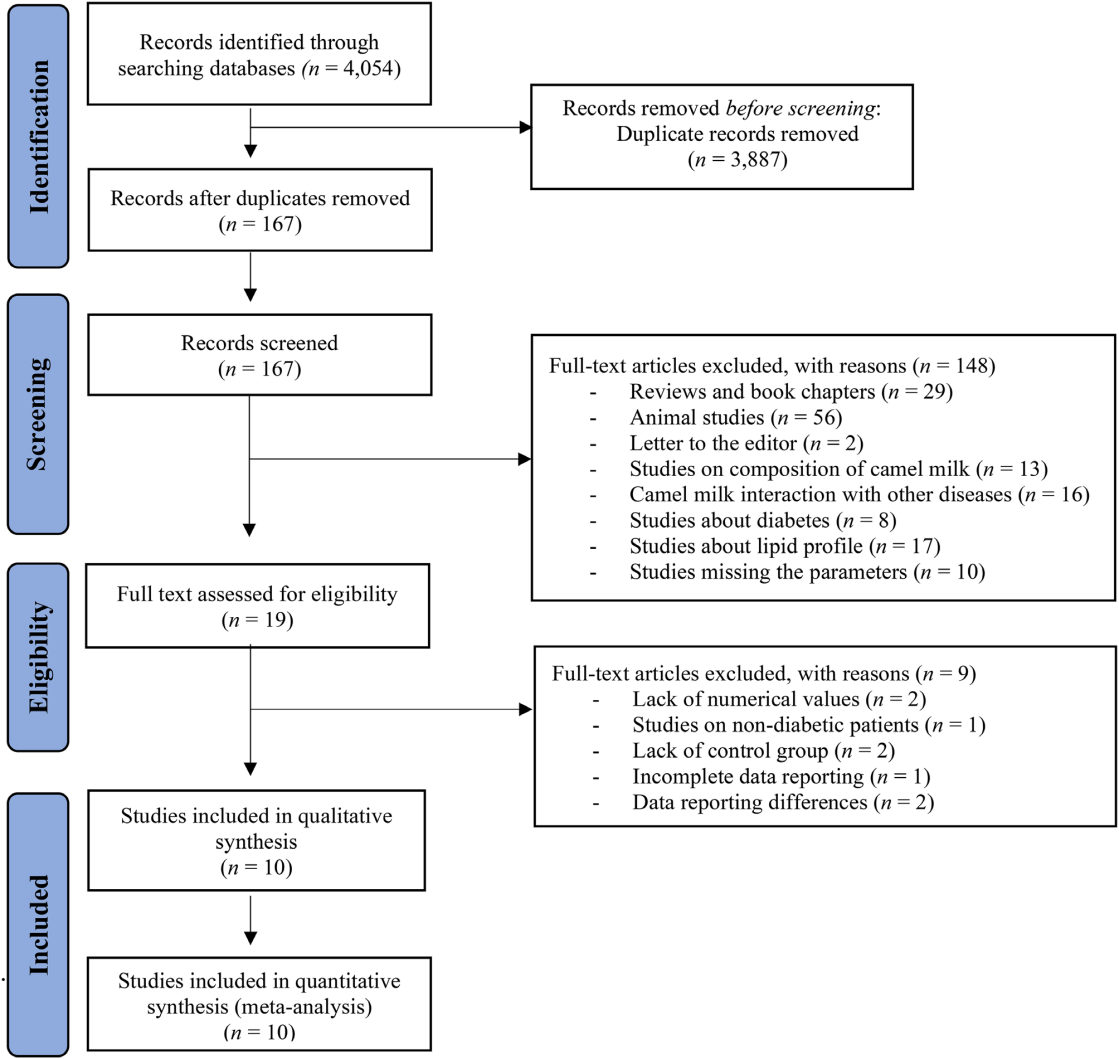
PRISMA flowchart diagram for study selection on the effect of camel milk on lipid profile in patients with diabetes

The primary search returned 4,054 studies (Fig. 1), of which 3,887 were deleted following duplicate checking. The remaining 167 studies underwent title and abstract screening, and 19 publications were retained for full-text screening. Nine studies were excluded because insufficient data were reported for the outcome measures of interest, leaving 10 studies [27, 33, 42–49] for inclusion in the quantitative meta-analysis and subgroup analyses.

### Characteristics of included studies

Table 2 presents the characteristics of the included studies. The sample sizes of these studies ranged from 12 to 64 participants (a total of 347 participants), and participants’ ages ranged from 8 to 70 years (excluding studies conducted only among patients aged < 18 years). One study was conducted among males alone [46], whereas the others included both sexes [27, 33, 42–45, 47–49]. Males comprised 60.5% of all participants in the included studies. The intervention duration ranged from 2 to 12 months, and the dose of CM given to the intervention groups ranged from 0.25 to 0.5 L daily or twice/week. The type of CM was described as fresh in 8 studies [27, 42–44, 46–49], fermented in one study [45], and pasteurized in one study [33]. The included studies were conducted in India [49], China [48], Egypt [27], Yemen [47], Iran [33, 42, 43, 45], Libya [46], and Sudan [44]. Six [27, 42, 44, 47–49] studies encouraged patients to follow exercise, diet, and insulin therapy 1 month before the intervention period.

**Table 2:** Characteristics and major findings of the included studies on the effect of camel milk (CM) on lipid profile in patients with diabetes

**Authors, publication year**	Country (city)	Sample size n (% male)	Mean age/age range (years)	Tested lipid profile component	Type of diabetes	Type of CM	Quantity of CM consumed (ml/day) by CM Group	Duration of intervention	Parameters of CM group		Parameters of the control group		Results (CM compared with control group)
									Before treatment	After treatment	Before treatment	After treatment	
Margdarinejad et al., 2021 [42]	Iran (Gorgan)	49 (44.9)	> 18	TC, TG	T2D	Fresh	500.0	≤ 6 months	TC: 158.21 ± 37.07 TG: 134.04 ± 94.98	TC: 157.04 ± 31.83 TG: 121.58 ± 46.86	TC: 169.80 ± 32.66 TG: 163.72 ± 68.03	TC: 168.60 ± 37.18 TG: 169.16 ± 73.47	CM and control groups: no significant difference in TC level. CM group: a significant decrease in TG.
Fallah et al., 2020 [43]	Iran (Tehran)	36 (36.1)	30–70	TC, TG, HDL, LDL	T2D	Fresh	500.0	≤ 6 months	TC: 157.50 ± 33.50 TG: 140.90 ± 84.40 HDL: 45.60 ± 8.10 LDL: 83.60 ± 21.00	TC: 164.60 ± 3.80 TG: 144.40 ± 10.10 HDL: 54.40 ± 2.90 LDL: 83.10 ± 4.00	TC: 163.20 ± 29.20 TG: 148.20 ± 20.40 HDL: 48.80 ± 18.00 LDL: 84.70 ± 5.10	TC: 152.80 ± 4.0 TG: 141.50 ± 18.90 HDL: 47.40 ± 3.10 LDL: 77.20 ± 6.00	Changes in lipid profile including TG, HDL, and LDL not statistically significant between CM and control groups.
Abdalla et al., 2018 [44]	Sudan (Al Qadarif)	30 (26.7)	8–19; Mean: 13.5	TC, TG, HDL, VLDL, LDL	T1D	Fresh	500.0	> 6 months	TC: 138.00 ± 52.00 TG: 100.00 ± 51.00 HDL: 63.00 ± 37.00 VLDL: 20.97 ± 10.05 LDL: 121.50 ± 56.50	TC: 89.50 ± 29.50 TG: 67.50 ± 27.50 HDL: 212.00 ± 72.00 VLDL: 13.50 ± 5.50 LDL: 27.00 ± 16.00	TC: 110.00 ± 55.00 TG: 85.00 ± 27.00 HDL: 103.50 ± 74.50 VLDL: 17.00 ± 5.60 LDL: 73.00 ± 41.00	TC: 130.00 ± 47.00 TG: 130.00 ± 32.00 HDL: 111.00 ±72.00 VLDL: 26.00 ± 6.70 LDL: 66.00 ± 34.00	CM: a significant decrease in TC (35%); LDL: reduced (78%); VLDL: reduced (33%); TG: reduced (33%); and a significant increase in HDL levels (236.5%). Control group: no improvement and no significant difference in lipid profile.
Fallah et al., 2018 [45]	Iran	24 (41.7)	11–18; Mean: 13.8	TC, TG, HDL, LDL	Pre-diabetes	Fermented	250.0	≤ 6 months	TC: 152.71 ± 30.21 TG: 119.17 ± 55.86 HDL: 41.08 ± 6.77 LDL: 85.13 ± 22.93	TC: 157.62 ± 44.64 TG: 128.58 ± 98.14 HDL: 40.08 ± 11.11 LDL: 89.38 ± 32.86	TC: 148.54 ± 25.72 TG: 115.13 ± 42.96 HDL: 40.42 ±7.60 LDL: 81.75 ± 19.97	TC: 152.62 ± 39.99 TG: 127.04 ± 82.23 HDL: 38.84 ± 12.50 LDL: 82.58 ± 31.08	CM: non-significant changes in lipid profile.
Shareha et al., 2016 [46]	Libya (Tripoli)	43 (100)	40–65	TC, TG	T2D	Fresh	500.0	≤ 6 months	TC: 171.76 ± 7.65 TG: 163.57 ± 4.30	TC: 168.24 ± 6.31 TG: 160.48 ± 3.97	TC: 180.32 ± 3.48 TG: 174.36 ± 4.40	TC: 179.32 ± 3.50 TG: 170.27 ± 4.40	CM group: TG significantly decreased; TC non-significantly decreased.
Ejtahed et al., 2015 [33]	Iran (Tehran)	20 (30)	20–70	TC, TG, HDL, LDL	T2D	Pasteurized	500.0	≤ 6 months	TC: 186.39 ± 39.06 TG: 139.95 ± 51.37 HDL: 47.18 ± 10.05 LDL: 106.34 ± 29.00	TC: 182.13 ± 42.92 TG: 139.95 ± 52.26 HDL: 50.27 ± 11.99 LDL: 103.25 ± 32.10	TC: 189.48 ± 32.10 TG: 153.23 ± 56.69 HDL: 49.11 ± 11.99 LDL: 104.02 ± 18.95	TC: 202.24 ± 54.14 TG: 174.49 ± 86.80 HDL: 50.27 ± 10.83 LDL: 117.17 ± 32.10	CM and control group: non-significant changes in lipid profile.
El-Sayed et al., 2011 [47]	Yemen	45 (66.7)	19–20	TC, TG, HDL, LDL	T1D	Fresh	500.0	≤ 6 months	TC: 251.80 ± 9.30 TG: 184.00 ± 2.20 HDL: 44.30 ± 2.00 LDL: 110.00 ± 2.90	TC: 209.20 ± 3.20 TG: 133.60 ± 4.20 HDL: 49.00 ± 1.50 LDL: 92.40 ± 2.60	TC: 271.80 ± 3.35 TG: 193.10 ±1.70 HDL: 43.10 ± 1.53 LDL: 109.90 ± 2.45	TC: 248.60 ± 3.70 TG: 175.70 ± 3.00 HDL: 43.70 ± 1.26 LDL: 102.60 ± 1.51	CM and control groups: a significant decrease in TG, TC, and LDL. HDL: significantly increased in the CM group only.
Mohamad et al., 2009 [27]	Egypt	64 (75)	17–20	TC, TG, HDL, VLDL, LDL	T1D	Fresh	500.0	≤ 6 months	TC: 265.70 ± 9.07 TG: 170.41 ± 21.60 HDL: 53.00 ± 12.60 VLDL: 14.50 ± 5.20 LDL: 103.84 ± 0.63	TC: 192.08 ± 11.04 TG: 157.20 ± 18.20 HDL: 50.70 ± 11.30 VLDL: 11.50 ± 3.90 LDL: 92.50 ± 17.80	TC: 266.20 ± 18.20 TG: 171.40 ± 21.60 HDL: 54.60 ± 12.50 VLDL: 14.40 ± 4.70 LDL: 99.60 ± 9.78	TC: 266.20 ± 18.20 TG: 171.40 ± 21.60 HDL: 54.60 ± 12.50 VLDL: 14.40 ± 4.70 LDL: 99.60 ± 9.78	CM: Significant decrease in TG only.
Wang et al., 2009 [48]	China (Beijing)	12 (83.3)	49–50	TC, TG	T2D	Fresh	500.0	> 6 months	TC: 297.76 ± 59.16 TG: 162.09 ± 33.66	TC: 235.89 ± 35.19 TG: 125.78 ± 83.26	TC: 286.16 ± 47.56 TG: 163.86 ± 37.20	TC: 278.42 ± 31.71 TG: 160.32 ± 34.54	TG and TC significantly decreased in the CM group.
Agarwal et al., 2003 [49]	India (Bikaner)	24 (83.3)	19–20	TC, TG, HDL, VLDL, LDL	T1D	Fresh^1^	500.0	≤ 6 months	TC: 164.58 ± 20.69 TG: 66.91 ± 25.60 HDL: 62.58 ± 13.91 VLDL: 13.50 ± 5.00 LDL: 92.00 ± 11.62	TC: 158.33 ± 21.55 TG: 60.16 ± 25.16 HDL: 66.66 ± 11.29 VLDL: 12.08 ± 5.08 LDL: 79.16 ± 17.75	TC: 165.83 ± 19.19 TG: 72.39 ± 20.71 HDL: 61.58 ± 9.10 VLDL: 14.41 ± 4.67 LDL: 89.58 ± 14.70	TC: 168.08 ± 15.61 TG: 72.00 ± 14.79 HDL: 58.66 ± 15.61 VLDL: 14.25 ± 3.16 LDL: 89.66 ± 12.26	CM: Significant decrease in LDL only.

Lipid profile: total cholesterol (TC, mg/dl), triglycerides (TG, mg/dl), high-density lipoprotein (HDL, mg/dl), very-low-density lipoprotein (VLDL, mg/d), and low-density lipoprotein (LDL, mg/dl)

^1^ The type of CM was not mentioned by the authors in this article, but it was counted as fresh based on previously published studies by the same authors

### Quality evaluation and publication Bias

**Fig. 2:**
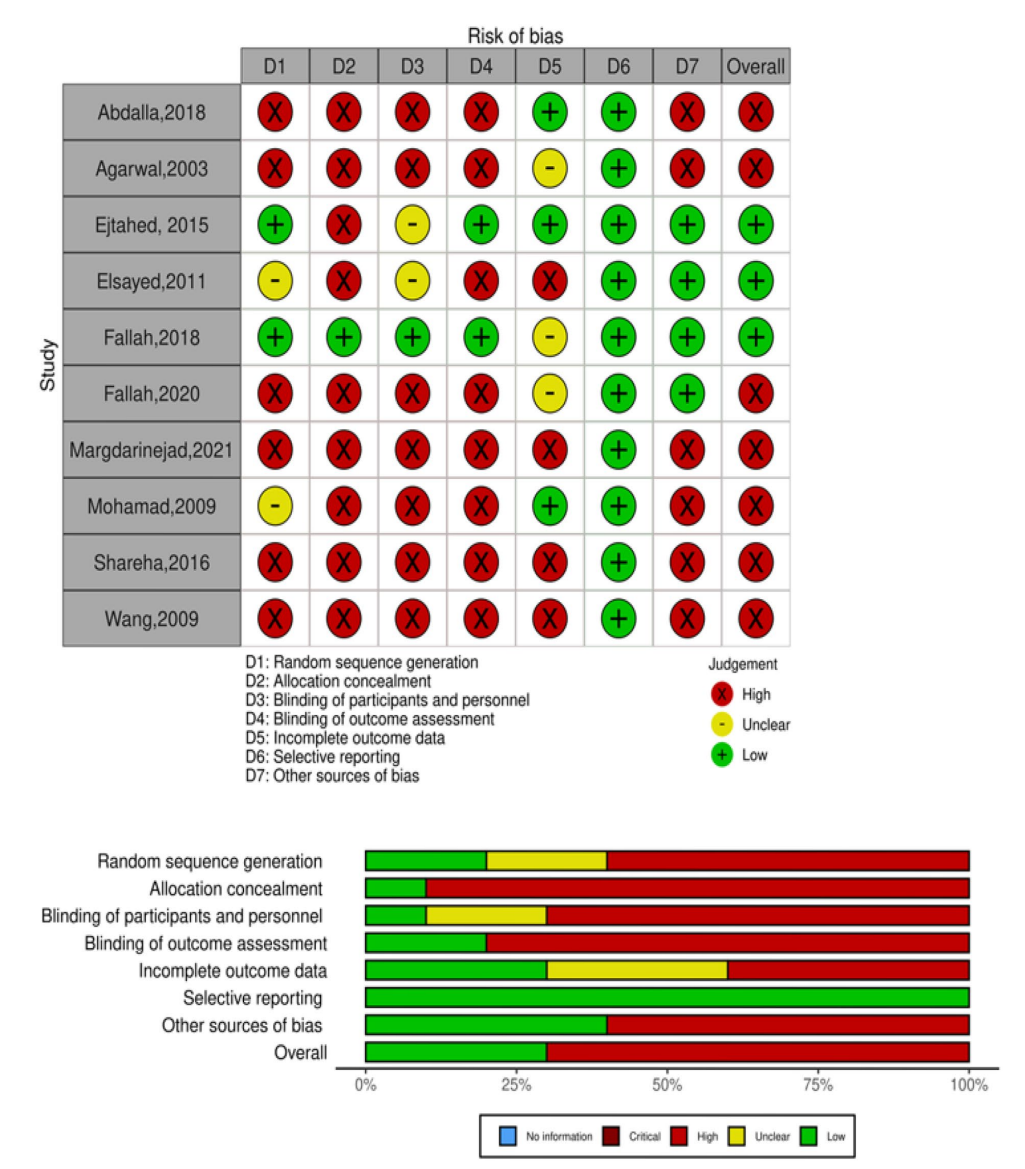
Summary of bias risk for each included study (N = 10); (b) risk of bias graph showing the percentage of bias risk for each included study. Green: low bias risk, Yellow: unclear bias risk, and Red: high bias risk

Figure 2 shows the risk of bias graph and summary plots. Sequence generation was performed adequately in two studies [33, 45]. One study had adequate concealment of participants’ allocation and adequate blinding of participants and key study personnel [45]. Two studies had a low risk of bias when blinding outcome assessment was used [33, 45]. Three studies adequately addressed incomplete outcome data [27, 33, 50] and there was a low risk of selective reporting bias in all included studies. Overall, 70% (7/10) of the included studies had a potential source of bias [27, 42, 43, 46, 48–50].

### Effect of CM intake on lipid profile

**Fig. 3:**
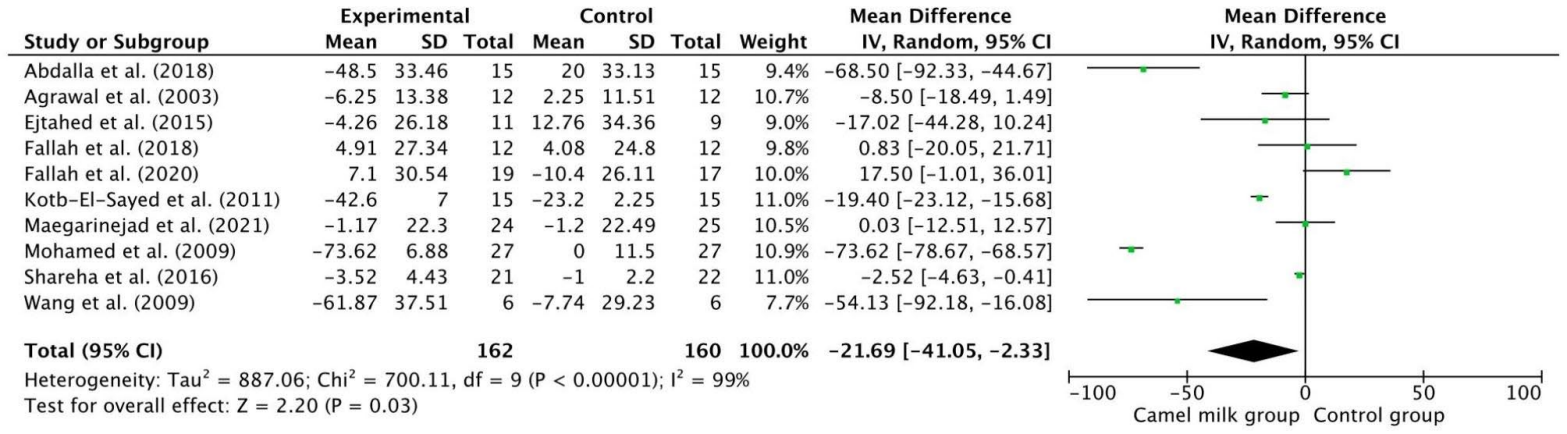
Forest plot for the effect of camel milk intake on total cholesterol (TC)

**Fig. 4:**
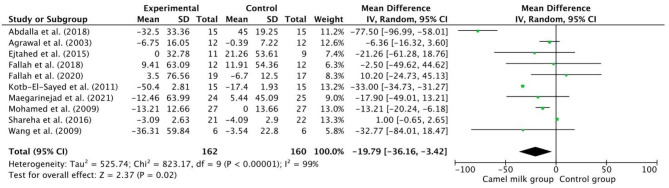
Forest plot for the effect of camel milk intake on triglycerides (TG)

**Fig. 5:**
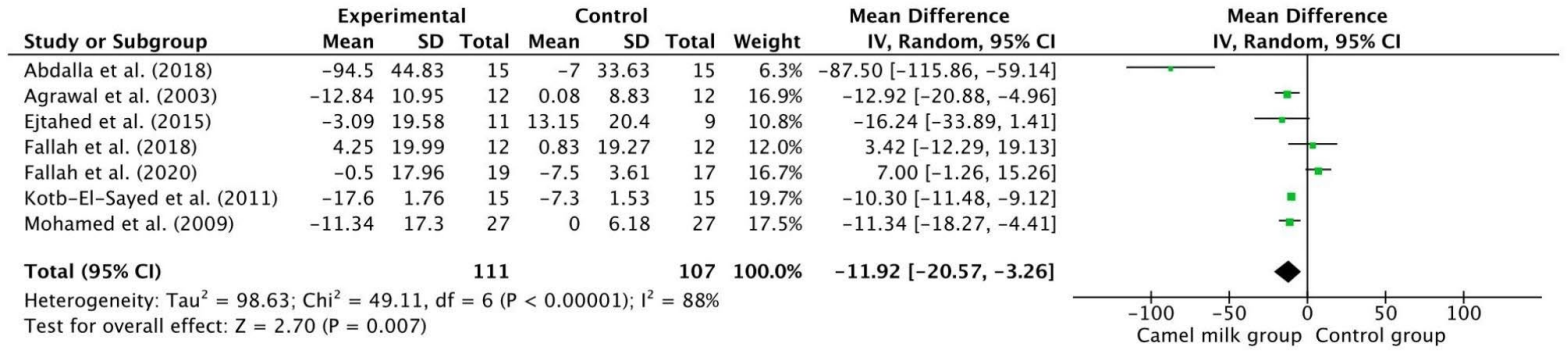
Forest plot for the effect of camel milk intake on low-density lipoprotein (LDL)

**Fig. 6:**
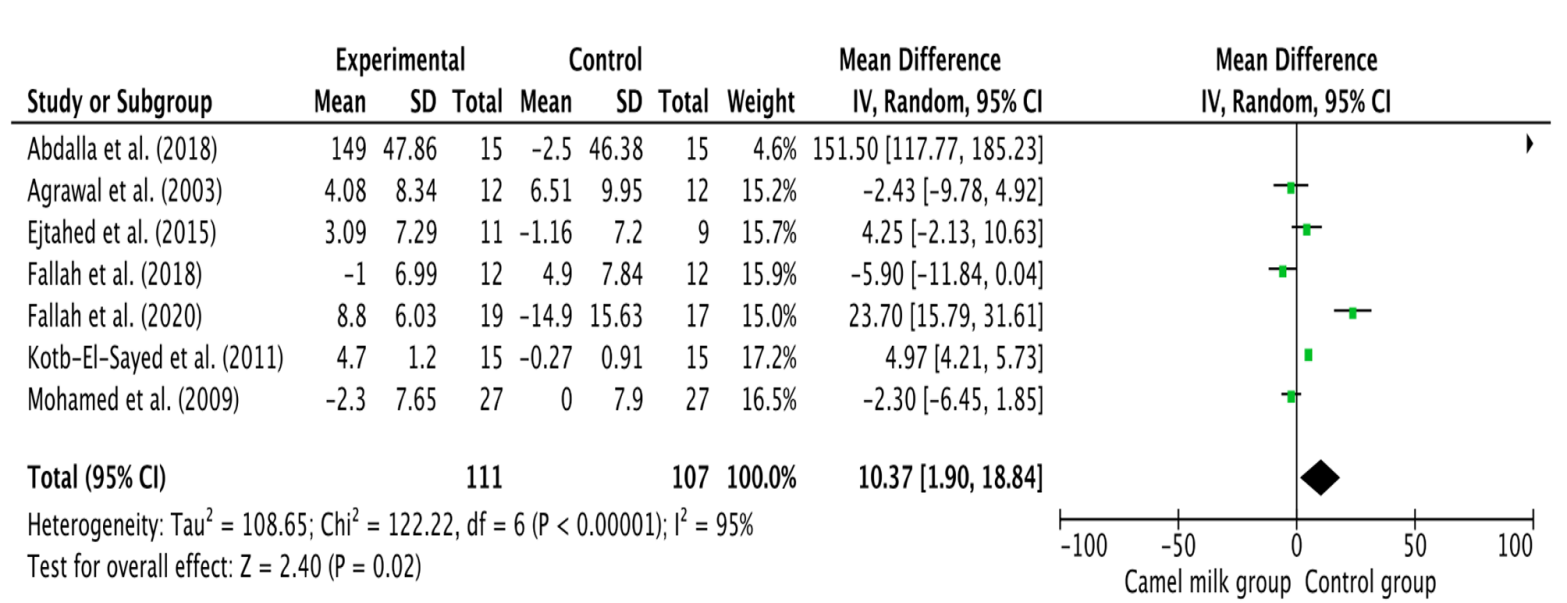
Forest plot for the effect of camel milk intake on high-density lipoprotein (HDL)

The pooled results from a random-effects model revealed that CM caused a statistically significant decrease in TC (MD − 21.69, 95% CI: 41.05, − 2.33; *p* = 0.03, *I*^2^=99%) (Fig. 3) and TG (MD −19.79, 95% CI: −36.16, −3.42; *p*=0.02, *I*^2^=99%) (Fig. 4) and LDL levels (MD −11.92, CI: −20.57, −3.26; *p* = 0.007, *I*^2^=88%) (Fig. 5) in patients who received CM compared with the control group. However, a non-significant decrease was reported in VLDL. On the other side, a significant increase in HDL levels (MD 10.37, 95% CI, 1.90, 18.84; *p*=0.02, *I*^2^=95%) (Fig. 6) was observed in patients supplemented with CM compared with the control group.

### Subgroup analysis

Subgroup analyses were conducted for lipid profile components that were reported in at least seven studies. Thus, subgroup analyses were performed only for TC, TG, LDL, and HDL because of the availability of studies. We stratified studies by the intervention duration (≤ 6 months or > 6 months), type of CM (fresh or treated such as fermented/pasteurized), and type of diabetes (T1D or T2D).

#### Intervention duration

Subgroup analysis revealed that only long-term interventions (> 6 months) elicited a significant reduction in TC levels (MD − 64.45, 95% CI: −84.65, − 44.26; *p* < 0.00001, *I*^2^=0%) (Supplementary Fig. 6) and TG levels (MD −61.67, 95% CI: −103.59, −19.75; *p*=0.004, *I*^2^=61%) (Supplementary Fig. 7).

#### Type of camel milk

Consumption of fresh CM by patients with diabetes resulted in significant reductions in TC (MD − 24.94, 95% CI: −46.69, − 3.19; *p* = 0.02, *I*^2^=99%) (Supplementary Fig. 8), TG (MD −20.94, 95% CI: −38.61, −3.27; *p*=0.02, *I*^2^=99%) (Supplementary Fig. 9), and LDL (MD −13.99, 95% CI: −24.23, −3.76; *p* = 0.007, *I*^*2*^ = 91%) (Supplementary Fig. 10) levels, while showed a significant increase in HDL levels (MD 17.80, 95% CI: 5.87, 29.72; *p* = 0.003, *I*^*2*^ = 96%) (Supplementary Fig. 11).

#### Type of patients with diabetes

Patients with T1D elicited a more pronounced effect in lowering TC (MD − 41.94, 95% CI: −77.10, − 6.79; *p* = 0.02, *I*^2^=99%) (Supplementary Fig. 12), TG (MD − 30.25, 95% CI: −48.17, − 12.34; *p*=0.0009, *I*^*2*^=96%) (Supplementary Fig. 13), LDL (MD − 19.13, 95% CI: −29.94, − 8.31; *p*=0.0005, *I*^*2*^=90%) (Supplementary Fig. 14) levels, and in increasing HDL (MD 15.83, 95% CI: 2.55, 29.11; *p*=0.02, *I*^*2*^=97%) (Supplementary Fig. 15) levels than those patients with T2D.

### Sensitivity analysis

When a sensitivity analysis was performed on articles that reported TC levels by removing one study at a time, the total effect size changed and became non-significant when the studies by Abdalla et al. [44], El-Sayed et al. [47], and Wang et al. [48] were excluded. However, the heterogeneity remained substantial. Consistent with the findings for TC levels, the sensitivity analysis for studies that reported TG showed that by eliminating the study by Abdalla et al., the overall effect became statistically non-significant, but the heterogeneity remained substantial. In the sensitivity analysis for HDL, the elimination of studies by Abdalla et al. and Wang et al. did not change the heterogeneity, but the overall effect became statistically non-significant. For VLDL, the overall effect became statistically significant after eliminating the study by Abdalla et al., and the heterogeneity was low (*I*^*2*^ = 25%). No changes were discovered in the data after performing a sensitivity analysis for LDL. Overall, there was a considerable level of heterogeneity among the studies.

## Discussion

To the best of our knowledge, this study is the first comprehensive systematic review and meta-analysis conducted to examine the impact of CM consumption on lipid profile as a key component of cardiometabolic health among patients with diabetes. In people with T1D and T2D, CM lowered TC, TG, and LDL while decreased VLDL little, and increased HDL. Total cholesterol, TG, and LDL levels decreased while HDL levels increased in diabetics who consumed fresh CM, according to subgroup analyses. However, only long-term therapies (> 6 months) reduced TC and TG levels significantly. Camel milk decreased TC, TG, and LDL and increased HDL in T1D patients more than in T2D patients. Camel milk’s lipid-normalizing actions supported its CVD preventive and treatment potential in patients with diabetes.

The potential of CM to improve blood lipids has been ascribed to various factors related to its composition, such as its fatty acid profile, which is known to be beneficial for human health [24]. It has been established that healthy, less saturated dietary fat choices are reflected in a greater intake of unsaturated fatty acids [51]; this lowers the chance of developing dyslipidemia, which is common among patients with diabetes [52]. Compared with bovine milk, which was used as a control in many of the RCTs included in our study, CM is richer in long-chain polyunsaturated fatty acids [53] and unsaturated fats (especially essential fatty acids linoleic and linolenic fatty acids) [54–56], and lower in cholesterol and saturated fats [57]. Furthermore, CM is known for its rich content of medium-chain fatty acids and mono- and polyunsaturated fatty (e.g., oleic and linoleic) acids, which have been shown to have beneficial effects in normalizing lipid profile and improving cardiometabolic health [23].

Another aspect of explaining the reported blood lipid-lowering effect of CM is the presence of conjugated linoleic acid (CLA), which enhances the ratio of plasma LDL to HDL via reducing TG levels [58]. The CLA in CM ranges between 1.2 and 1.5% of the total fat content and varies depending on the source, diet, stage of lactation, and management system [59]. These differences may play a role in the variations in responses of patients with diabetes to the consumption of CM and the outcomes on blood lipids. Camels that consume a high-forage diet tends to have higher CLA content in their milk than camels on high grains diets. CM also has a higher CLA content than cow’s milk, which typically contains 0.2% CLA, because camels can convert linoleic acid (a type of omega-6 fatty acid) into CLA [23]. The high L-carnitine concentration in CM may also be beneficial for the lipid profile, either directly by inhibiting the absorption of exogenous cholesterol or indirectly by enhancing the transportation of long-chain fatty acids to mitochondria for catabolic β-oxidation [60–62]. This may enhance adipose tissue weight loss [63], which in turn has beneficial effects on the lipid profile.

A different aspect is the unique proteins in CM (e.g., insulin-like peptides and lactoglobulins) which are known to induce positive impacts on glucose and cholesterol levels and may contribute to its ameliorating effects on cardiometabolic markers and lipid profile in particular [64–67], and helps in reducing the needed amount of exogenous insulin in individuals with T1D [68, 69]. Insulin has a fundamental metabolic effect in regulating blood lipids, which explains why patients with diabetes have dyslipidemia as a coexisting metabolic condition [70]. Lipid and glucose profiles are the most important indices for patients with diabetes, and there are strong correlations with abnormalities in these profiles [71]. Because of the presence of these insulin-like peptides, CM has the ability to normalize glucose homeostasis by decreasing the amount of insulin dose needed improving insulin sensitivity, and decreasing insulin resistance [16]; this could also explain why CM may normalize blood lipids and lower blood cholesterol in patients with diabetes.

Orotic acid (OA), also known as orotate, is naturally present in foods (particularly milk and dairy products) and is recognized as a precursor in the biosynthesis of pyrimidines. The body converts OA to uridine, which is used in the pyrimidine salvage pathway. This conversion primarily occurs in the liver, kidney, and erythrocytes. OA was initially labeled “vitamin B13,” and its application in combination with organic cations or metal ions gained popularity in fields such as body-building and the treatment of metabolic disorders [72]. CM contains OA, and several animal and human studies have indicated that OA may be involved in lowering cholesterol [24, 65, 66, 73, 74].

The present findings drawn from human studies were consistent with prior research in animal models that demonstrated CM intake had beneficial effects in improving lipid profiles of chemically-induced diabetes. CM was reported to decrease TC, TG, and HDL cholesterol [75], and may also decrease the synthesis of hepatic cholesterol [76]. In a study involving rabbits, CM significantly reduced TC, TG, and HDL [75], with this cholesterol-lowering effect ascribed to improvement in the body’s oxidative status via a reduction in the catalase and peroxidase enzymes. Increased fecal excretion of cholesterol, improved hepatic glutathione peroxidase, and attenuated hepatic thiobarbituric acid were other plausible mechanisms for the lipid-lowering effect of CM demonstrated in animal models [75, 77]. Another study showed significant improvements in TC, TG, LDL, and VLDL levels in 20 male Wistar rats after exposure to CM [78]. In that study, the atherogenic index dropped dramatically in the group receiving a high fat, cholesterol-rich diet plus CM compared with the group without CM, indicating CM consumption had a beneficial anti-atherosclerosis effect in animals [78].

Recent in vivo research suggested the cardio-preventive effects of fermented CM may be attributable to the inhibition of CCl4-induced toxicity [79], and administration of fermented CM to adult male Wistar rats significantly reduced serum cholesterol levels and the atherogenic index [80]. Consumption of CM also dramatically decreased TC and TG levels and liver enzymes (ALT and AST) among adult female Albino rats [81].

### Subgroup analysis

#### Type of diabetes

The reported more pronounced effect of CM on patients with T1D in normalizing blood lipids is consistent with findings of our meta-analysis on the effect of CM on glucose homeostasis in patients with diabetes, where the insulin dose for patients was significantly decreased by the consumption of CM (MD, − 16.72, 95% CI: −22.09, − 11.35 *p* < 0.00001, *I*^*2*^ = 90%) in comparison with the controls [16]. Furthermore, the latter meta-analysis revealed that CM exhibited a more pronounced positive effect in lowering HbA1c in individuals with T1D than in those with T2D. The concept of having “insulin-like peptides” aids in elucidating the notion of the notable improvement in lipid profile observed in individuals with diabetes who receive insulin injections [82]. This assertion is more corroborated by the clinical investigation conducted on the ingestion of CM in individuals diagnosed with T1D. The findings of one study revealed that regular consumption of CM resulted in a significant reduction in fasting blood glucose levels and a drop in the average insulin dosage required by 37% (from 30.40 ± 11.97 to 19.12 ± 13.39 units per day) [83]. Nevertheless, a recent analysis of insulin immunoreactivity in CM samples indicated a deficiency of insulin in significant amounts (falling below the detectable range using the anti-human insulin antibody). Consequently, it is inferred that the blood lipid-improving impact of CM may be attributed to components other than insulin-like peptides [84]. In that essence, the multiplex panel analysis revealed that the CM samples exhibited the presence of insulinotropic polypeptide (also known as gastric inhibitory polypeptide, GIP) and showed elevated immunoreactivity towards visfatin, resistin, and ghrelin compared to the other ruminant milk samples that were analyzed [84]. Most recently, detailed mechanistic and molecular insights on CM revealed that the peptides from CM with anti-diabetic properties, which are mainly produced through bacterial fermentation and enzymatic hydrolysis, aid in the noticed improvement in lipid profile exerted by CM consumption [85]. Regarding T2D and lipid profile, it appears that there’s still a paucity in the existing literature about the impact of CM on T2D, with contradicting findings that warrant executing further randomized controlled trials.

#### Intervention duration

The reported superiority of long-term intervention (> 6 months) over short-term (< 6 months) in normalizing blood lipids is consistent with our previous meta-analysis showing that HbA1c showed a superior and more significant decrease among those patients who received CM for long duration than those on short duration [16]. This superiority could be explained by the fact that consistent and repeated exposure to the bioactive peptides of CM would exaggerate and accumulate the positive effects induced by these peptides on the lipid profile.

#### Type of CM

The dyslipidemia-preventive effect induced by CM could also be attributed to the bioactive peptides found in CM, as said before [29], especially when CM undergoes fermentation [66]. However, the present study revealed that raw CM had a greater positive effect than treated (fermented/pasteurized) CM. This contradicting result could be because the small number of studies meant we merged two types of treatments (fermentation and pasteurization) into one group, and the positive effect of fermentation could therefore have been masked by heat treatment (pasteurization), which negatively affects the activity of the CM bioactive peptides. The interaction between these bioactive peptides/proteins and cholesterol decreases cholesterol levels, as shown in many studies [24, 29].

Thermal and non-thermal treatments for CM affect its nutritional, biological, microbiological, and functional properties [86], which could in turn impact its potential as a blood lipid-lowering agent. The present study found untreated, fresh CM had a superior effect on lipid metabolism regulation among patients with diabetes compared with treated (pasteurized/fermented) forms However, few studies have investigated the effects of various treatments on CM and lipid metabolism. The noted superiority of fresh over treated CM in improving the lipid profile in the current work could be attributed to the negative effects of heat treatment on the functional properties of CM peptides, which could be summarized as follows [86, 87]: (i) denaturation of proteins or changes in the distinct three-dimensional configuration can lower their ability to act as functional peptides in normalizing blood lipids, and (ii) changes in the bioactivity of CM functional peptides. That is, heat treatment may alter the bioactivity of the functional peptides present in CM such as causing changes in the conformation of enzymes, reducing their activity and effectiveness as functional peptides, and (iii) destruction of functional peptides, in which heat treatment may destroy functional peptides, which in turn can reduce the overall concentration of these bioactive peptides in CM, making it less effective as a functional ingredient in lowering blood lipids. Finally, it is important to note that the extent to which heat treatment affects the functional properties of CM peptides depends on the temperature, duration, and processing conditions [86, 87]. In general, gentle processing methods and low heat treatment temperatures are recommended to preserve the functional properties of CM bioactive peptides [29, 87–89].

### Study strengths and limitations

The present study had several strengths, including being the first meta-analysis in this field to evaluate multiple parameters related to CM consumption and lipid profile among patients with diabetes. The analysis was stratified by the type of diabetes, type of CM, and intervention duration. However, some limitations need to be considered when interpreting our results. The selected studies had significant methodological and statistical differences, which could be attributable to various factors such as differences in intervention duration, type and amount of CM used, type of diabetes, medications used, participants’ age and sex, and time since diabetes diagnosis. This highlights the need for further RCTs with standardized study components to better understand the impact of CM on lipid profiles and minimize the impact of confounding factors. Given the evidence for the lipid-improving effects of CM, consumption of CM as part of regular meals may be a useful adjuvant therapy for patients with T1D or T2D. This could lower treatment costs for dyslipidemia-characterized patients with diabetes and help reduce the need for lipid-lowering medications, leading to fewer long-term potential side effects. More mechanistic research is needed to fully understand and elucidate the mechanisms underpinning how CM can improve lipid profile, especially among patients with diabetes.

## Conclusion

Our findings suggest that CM *could* be a beneficial *complementary* treatment in the context of dyslipidemia management needed for patients with both T1D and T2D, in terms of its ability to decrease blood TC, TG, and LDL, and increase HDL levels. Long-term consumption (> 6 months) of CM by patients with diabetes *may be a helpful adjuvant therapy* alongside the prescribed drugs for improving lipid profile, particularly in patients with T1D. However, because of the observed *high heterogeneity* in the included studies, further well-designed RCTs employing larger sample sizes and longer durations are needed to confirm these findings and provide more robust evidence of the impact of CM on the lipid profile of patients with diabetes.

### Electronic supplementary material

Below is the link to the electronic supplementary material.

Supplementary Material 1

## Data Availability

Data available on request to the corresponding author because of restrictions (e.g., privacy or ethics).
