# Supplementary material for: Effect of camel milk on lipid profile among patients with diabetes: a systematic review, meta-analysis, and meta-regression of randomized controlled trials
Source: BMC Complement Med Ther. 2023 Dec 4;23:438. doi: 10.1186/s12906-023-04257-5 (PMC10696884; doi:10.1186/s12906-023-04257-5)
Supplement: Supplementary file 1 — Supplementary Material 1 [file 12906_2023_4257_MOESM1_ESM.docx]

<https://drive.google.com/file/d/1md9WCLkzULGHhaXEvchYtHdR3ybCHask/view?usp=sharing>
